# A particle swarm optimization-based algorithm for finding gapped motifs

**DOI:** 10.1186/1756-0381-3-9

**Published:** 2010-12-13

**Authors:** Chengwei Lei, Jianhua Ruan

**Affiliations:** 1 Department of Computer Science, The University of Texas at San Antonio, San Antonio, TX 78249, USA

## Abstract

**Background:**

Identifying approximately repeated patterns, or motifs, in DNA sequences from a set of co-regulated genes is an important step towards deciphering the complex gene regulatory networks and understanding gene functions.

**Results:**

In this work, we develop a novel motif finding algorithm (PSO+) using a population-based stochastic optimization technique called Particle Swarm Optimization (PSO), which has been shown to be effective in optimizing difficult multidimensional problems in continuous domains. We propose a modification of the standard PSO algorithm to handle discrete values, such as characters in DNA sequences. The algorithm provides several features. First, we use both consensus and position-specific weight matrix representations in our algorithm, taking advantage of the efficiency of the former and the accuracy of the latter. Furthermore, many real motifs contain gaps, but the existing methods usually ignore them or assume a user know their exact locations and lengths, which is usually impractical for real applications. In comparison, our method models gaps explicitly, and provides an easy solution to find gapped motifs without any detailed knowledge of gaps. Our method allows the presence of input sequences containing zero or multiple binding sites.

**Conclusion:**

Experimental results on synthetic challenge problems as well as real biological sequences show that our method is both more efficient and more accurate than several existing algorithms, especially when gaps are present in the motifs.

## Background

Computational prediction of transcription factor binding sites (TFBS) from co-expressed/co-regulated genes is an important step towards deciphering complex gene regulatory networks and understanding gene functions. Given the promoter sequences of a set of co-expressed/co-regulated genes, the goal is to find short DNA sequences ("motifs") whose occurrences (with allowed mismatches) in the sequences cannot be explained by a background model. An accurate identification of such motifs is computationally challenging, as they are typically very short (8-15 bases) compared to the promoter sequences (hundreds to thousands bases). Furthermore, there is often a great variability among the binding sites of any given TF, and the biological nature of the variability is not yet well understood. Finally, in many cases, the TFBS may appear only in a subset of the putatively co-regulated genes.

Despite the challenge, many computational methods have been developed and have been proven useful in predicting real binding sites [1]. The existing algorithms can be roughly classified into two broad categories according to the motif representations: those based on position-specific weight matrices (PWMs), and those based on consensus sequences. Examples of the former include well-known programs such as MEME [2], AlignACE [3], GibbsSampler [4], and BioProspector [5]. The latter category includes Weeder [6], YMF [7], MultiProfiler [8], and Projection [9]. In general, PWM offers a more accurate description of motifs than consensus sequences, but the score of PWM is more difficult to optimize. On the other hand, consensus-based algorithms often rely on enumerating short subsequences, which may be impossible for longer motifs. For an excellent survey of the existing methods and an assessment of their relative performance, see [1,8].

Recently several consensus-based motif finding algorithms have been developed using evolutionary algorithms, because of their efficiency in searching over multidimensional solution spaces. For example, GAME [10] and GALFP [11] are based on genetic algorithms, and have been shown to outperform many PWM-based algorithms. In a previous work, we proposed a motif finding algorithm based on the classical Particle Swarm Optimization (PSO) strategy [12], where we used the set of positions on each sequence together as a solution, and searched the solution space by PSO algorithm. To keep the solution space continuous, we restructured the original sequences using a sequence mapping. Although the algorithm shows a good performance on small input size (for example 20 sequences and 1000 bases for each sequence), the algorithm becomes slow for larger data sets, as the number of possible motif positions grows exponentially as the number of sequences increases. Several other motif finding methods have also been developed based on PSO, for example, Hybrid-PSO [13] and PSO-EM [14]. Hybrid-PSO uses a similar basic idea as our previous work [12], and therefore has the same problem we mentioned above. PSO-EM simply uses PSO to find candidate motifs, which are then used as seeds by other expectation-maximization based motif finding algorithms, such as MEME [2].

In this paper, we develop a novel algorithm, called PSO+, for finding motifs. This new method has the following contributions. First and most importantly, PSO+ differs from other motif finding algorithms by explicitly modeling gaps, which provides an easy solution to find gapped motifs. Many real motifs contain positions of low information (gaps), but the existing algorithms usually do not allow gaps, or require a user to specify the exact location and length of gaps, which is often impractical for real applications. Second, we use both consensus and PWM representations in our algorithm, taking advantage of the efficiency of consensus and the accuracy of PWMs. Our method also allows some input sequences to contain zero or multiple binding sites, which is common in real biology data set, but ignored by some of the algorithms. Finally, we propose a novel modification to the PSO update rule to accommodate discrete values, such as characters in DNA sequences, which may also be useful in other applications.

## Methods

### Introduction to Particle Swarm Optimization

Particle Swarm Optimization (PSO), which has been shown to be effective in optimizing difficult multidimensional problems in many fields, is a population-based stochastic optimization technique for problem solving that is inspired by the social behaviors of organisms such as bird flocking [15]. The system is initialized with a population of random solutions and searches for the optimal solution by updating iteratively. Each potential solution, called particle (or agent), is represented by a point in the multiple-dimensional solution space. When searching for the optimum solution, particles fly around the solution space with a certain velocity (speed and direction). During flight, each particle adjusts its position and velocity according to its own experience and the experience of its neighbors. Specifically, each particle keeps track of the best solution it has encountered so far. This solution is called *pbest*, which stands for personal best. The system also keeps track of the global optimum of all the particles, hence called *gbest*. The fundamental concept of PSO consists of changing the velocity of each particle at each time step toward its *pbest *and *gbest *locations [15].

### Method overview

**Figure 1:**
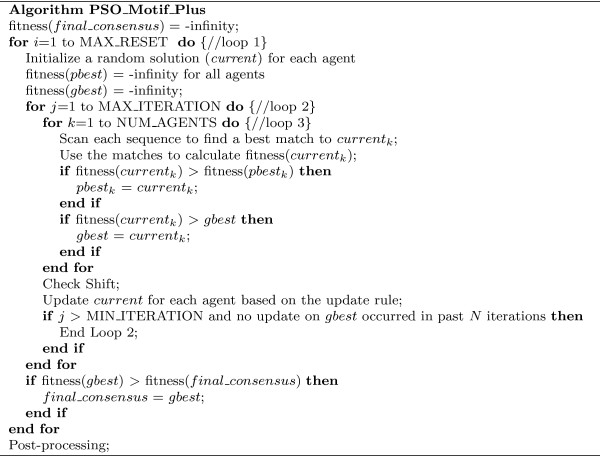
**Pseudo-code**. Pseudo-code of our PSO+ algorithm.

Figure 1 shows the main structure of the algorithm, which contains three loops. The most inside loop, loop3, evaluates the fitness value of each agent and updates information for the whole system. Using its *current *solution, an agent first finds out a best match from each sequence, calculates the fitness value (see below), and updates *pbest*, *gbest *if necessary. Loop2 is the main part of the PSO+ algorithm. From a random initial solution, each agent continuously searches for better solutions in the neighborhood, taking information from its own experience (*pbest*), and the experience of all agents (*gbest*). The actual movement of each agent is determined by the update rule (see below). Finally, as a stochastic algorithm, the final solution of PSO+ depends on its starting solutions; the purpose of loop1 is therefore to restart the system several times, from independent random solutions, to ensure a high overall success rate. At the final step, we use post-processing to remove and/or add some binding sites, therefore allowing zero or multiple binding sites on each sequence. A flow chart is shown in Additional file 1 Figure S1.

### Solution space and fitness function

To utilize the PSO+ algorithm, we need to represent a solution as a vector, and determine a fitness function appropriate for the problem. As discussed in Background, a motif of length *l *can be represented either as a position-specific weight matrix (PWM), which is a 4×l matrix of real numbers specifying the probability of each base at each position, or a consensus describing the most dominate base at each position. The matrix can be converted into a vector of length 4*l*, although some care needs to be exercised to ensure proper normalization. The consensus representation is more efficient in searching for new instances, and may lead to faster convergence, while the PWM is more powerful in representing weaker motifs and is more accurate in evaluating the motif quality. We decided to use both representations in our algorithm, to take advantage of both forms. The solution is initialized as a consensus. During the scanning stage, we use the consensus representation. After the best matches to the consensus are found from all the sequences, we compute a PWM based on the set of matches, and compute the fitness of the solution based on the PWM.

Given a solution, i.e., a consensus, it is first used to scan the input sequences to find a best match on each sequence. Let *X *= (*x*_1 _*x*_2 _... *x_l_*) be the consensus sequence and *Y *= (*y*_1 _*y*_2 _... *y_l_*) be a putative binding site.

The matching score between *X *and *Y *is computed using the following equations:

$$\begin{array}{c} M(X,Y)=\sum_{i}\sigma (x_{i},y_{i})\;\text{and}\\ \sigma (a,b)=\{\begin{array}{cc} 1+\log_{4}(0.25/p_{a}) & \text{if}\;\;a=b\\ \log_{4}(0.25/\sqrt{p_{a}p_{b}}) & \text{otherwise} \end{array} \end{array}$$

where *p_a _*is the background frequency for base *a *in the input sequences or in the whole genome. As most genomes contain more AT's than CG's, this formula gives unequal weight to different types of matches/mismatches. A match between two bases with lower background frequency would have higher score than that between two bases with higher background frequency. For uniform base frequency *p_A _*= *p_C _*= *p_G _*= *p_T _*= 1/4, *σ *(*a*, *b*) = 1 if *a *= *b *and 0 otherwise, corresponding to an intuitive match/mismatch score.

Given a set of matches of a consensus, *W *= *w*_1_, *w*_2_, ..., *w_n _*where each *w_i _*is a subsequence with length *l *, we compute the fitness of the consensus using the information content (IC) score:

$$IC\;=\sum_{j=1}^{l}\sum_{b}f_{b}(j)\log_{2}(f_{b}(j)/p_{b}),$$

where *f_b_*(*j*) is the normalized frequency of nucleotide *b *on the column *j *of all instances in *W *and *p_b _*is the background frequency of *b*.

### Initial solutions and number of agents

Similar to all stochastic algorithms, the performance of PSO+ partially depends on the initial solutions. The convergence of the algorithm can be significantly improved if at least one of the agents has an initial solution near the optimal solution. In this work we consider two strategies. The first strategy is to simply generate a set of random consensus. The second strategy is to randomly choose a subsequence from the input sequence as an initial solution. Although the first strategy would allow the maximum coverage, the probability that any randomly generated consensus is near the optimal solution is very low, as there are 4*^l ^*possible solutions for a motif of length *l*. For the second strategy, we assume that the actual binding site is closer to the consensus than to random sequences. Since there is usually about one binding site per sequence, it is very likely that some agents may select a binding site as an initial solution. More precisely, assuming that the average sequence length is *L *and the motif length is *l*, the probability that a randomly selected sequence is a binding site is 1/(*L *- *l *+ 1). Also because of the Check Shift step in the algorithm, a random solution that contains a large suffix or prefix of the binding site can often lead to the recovery of the real motif quickly. We usually allow a binding site to be shifted by two bases to its left and right, respectively. Therefore, each true binding site can provide up to 5 initial solutions that are similar to the real motif. For this reason, we suggest the minimum number of agents to be (*L *- *l *+ 1)/5 to ensure a high convergence probability. In our experiments on both synthetic and real sequences, we have found that the second strategy usually leads to much faster convergence and therefore is implemented as the default option.

### Modified PSO+ update rule for discrete problems

After each iteration, each agent needs to update its *current *solution based on the old *current*, its own *pbest*, and *gbest *of the system, each of which is a vector. The standard PSO algorithm is designed for optimization problems in the continuous domain; therefore, a new solution can be easily obtained by a sum of the three solution vectors multiplied by some random weights. In our case, however, each solution is a vector of ACGT's and they cannot be manipulated by multiplication and summation. In order to generate a new solution, we use the following rule, which is applied independently to each position of the motif:

$$i*=\arg\max_{i}(c_{i}r_{i}\;\text{weight}(x_{i}))\;\text{and}\;x^{'}=x_{i*},$$

where *x' *is the new character being generated, *x*_1_, *x*_2_, *x*_3 _are the characters in *current*, *pbest*, and *gbest*, respectively, *x*_4 _is a random character from ACGT, *c_i _*is a scaling factor to determine the relative importance of the four terms, *r_i _*is a uniform random number, and the function weight gives a higher weight to characters having lower background frequency. This strategy effectively suppress characters with lower occurrence, when compared to an alternative strategy that randomly picks a character proportional to its number of occurrences in *x*_1_, *x*_2_, *x*_3 _and *x*_4_. For example, assuming *c_i _*= 1 and the weight function returns a constant, if *x*_1 _= *x*_2 _= *x*_3 _= A and *x*_4 _= C, the alternative strategy would select A with 3/4 probability and C with 1/4 probability; with our strategy, we would select A with 7/8 probability and C with 1/8 probability. This reduces the probability of drifting away when a solution is near the optimal solution.

All agents stop their movements if the number of iterations exceeds an upper limit, or if there has been no update for a certain number of iterations and a minimum number of iterations has passed.

### Check shift

Similar to many motif finding algorithms, the output of PSO+ algorithm may have a shift issue: the start positions of the binding sites may be one or two positions away from the real position, and it is difficult for the algorithm to escape from such local optima. To circumvent this problem, we periodically check whether shifting the binding sites by a small number (up to 3 bases) can improve the quality of the solution. This is done in the CHECK SHIFT step.

### Gapped motifs

Many real motifs contain *do-not-care *positions in their consensus. Furthermore, these *do-not-care *positions are often consecutive, forming a motif with two short conserved regions with some fixed distance in between. We call these *do-not-care *positions gaps and the motifs gapped motifs. Most motif finding algorithms do not consider gaps explicitly. For consensus-based algorithms, ignoring gaps can lead to serious mistakes when a consensus is forced to be selected for a gap position, which has no dominant characters.

#### Gap representation

We solve this problem by asking the users to provide two parameters, motif length *l*, and gap length *k*. Importantly, when we search for gapped motifs, we allow gaps to appear as the suffix or prefix of a motif. This is very useful if the actual gap is shorter than *k*, or if the non-gap region of the motif is shorter than *l *- *k*. Furthermore, if a real motif contains no gaps, our algorithm will automatically put all gaps in the flank regions. While our algorithm may increase the guesswork from the user by asking for gap length as an additional parameter, it actually gives the user more flexibility in determining the appropriate motif and gap lengths.

To find gapped motifs, we introduce a bit vector of length *l*. The bit vector contains exactly *k *0's and *l *- *k *1's, where a 0 means the position falls in a gap. This vector is initialized randomly and latter updated by masking out the columns in the PWM with low IC values. Given this vector, when calculating the match score between a consensus and a potential binding site, we only consider the positions with 1's. When we compute the fitness (IC score) of a motif given all the binding sites, we consider all columns, because in this case we have all the information to derive a PWM.

#### Gap insert strategy

We consider two types of gaps. The first type of gaps can be anywhere in the motif and do not need to be consecutive. To find this type of gapped motifs, we simply mark the columns with the lowest IC value as gaps. The second type of gaps are consecutive and are located in the center of a motif. For this type of gapped motifs, we require a motif to contain at most two consecutive non-gapped regions, while gaps can appear either as a prefix, a suffix, or in the center of the motif. An algorithm using the second type of gaps is less efficient, but can often result in better results for real motifs. This is the default option of our algorithm. Experimental results to support this default option are shown in Additional file 1 Table S1 and Table S2.

#### Flexible gap length

If the actual gap is shorter than the given number, or if the motif is shorter than expected, gaps can appear as suffix and/or prefix of the motif. Furthermore, the algorithm will automatically put all gaps in the flank regions if the real motif contains no gaps.

By default, we use *8-base motif with 0 gap *for short motifs, *12-base motif with 2 gaps *for medium-length motifs, and *16-base motif with 4 gaps *for long motifs. This strategy works well for the unknown length motif finding problems in this paper. The program allows a user to change the parameters by themselves.

### Post-processing

The basic algorithm described above assumes that there is one and only one binding site on each sequence, which is certainly not always true. To address this problem, we use a statistically-inspired strategy to refine the binding sites. We assume that at least a good fraction of the sequences contain at least one binding site. We calculate the match score for each putative binding site returned from the basic algorithm. Let Q1, Q2, and Q3 represent the lower quartile, median, and upper quartile of the match scores. The inter-quartile range (IQR) of the match scores is then computed by Q3 - Q1. All binding sites with a match score below Q1 - IQR are dropped as false binding sites. We also rescan the input sequences using the consensus for additional putative binding sites. A binding site with match score higher than Q2 is considered a true binding site. A PWM is constructed using the final set of binding sites.

## Results and discussion

To evaluate the performance of our algorithm, we tested it on two types of sequences. The first type of test data consists of synthetic DNA sequences, also known as the (l, d)-motif challenging problem [16]. The second type of data contains real promoter sequences. The algorithm is implemented in C. The test datasets are included in Additional file 2.

### Evaluation using synthetic data sets

We tested our algorithm on the (*l*, *d*)-motif challenge problem. Each challenge problem includes *n *sequences of length *L*, each of which contains a variant of a pre-defined consensus of length *l*. The variants were generated by choosing *d *positions randomly from the consensus and changing them to random bases.

In our first experiment, we focused on the (15, 4)-motif challenge problem, which is one of the most popular benchmarks for motif finding programs. We chose *n *= 20, and varied *L *from 400 to 1000. Table 1 (left half) shows the running time of our current algorithm (PSO+), another PSO-based algorithm we developed recently (PSO) [12], two evolutionary algorithms (GAME [10] and GALFP [11]), and three well-known combinatorial-search algorithms (Projection [9], Weeder [6], and MotifEnumerator [17]). We were not able to test Weeder by ourselves because the currently available implementation of the algorithm can only handle motifs of even lengths up to 12. Its running time was taken from the original publication and was based on an 89% success probability [6]. The results of the other algorithms were obtained by downloading the programs from the original authors' websites and running with parameters that can recover the embedded motifs with 100% accuracy. Running time was based on the average of 10 runs on 5 sets of sequences.

**Table 1:** Running time (seconds) on (l, d)-motif challenge problems

Sequence length	400	500	600	800	1000	600	600	600	600
**(*l, d*)**	**(15,4)**	**(15,4)**	**(15,4)**	**(15,4)**	**(15,4)**	**(11,2)**	**(13,3)**	**(17,5)**	**(19,6)**

Weeder	60	125	200	450	900	-	-	-	-
Projection	9	23	42	162	418	4	13	94	174
MotifEnumerator	-	-	-	-	-	5	119	-	-
PSO	18	34	57	137	288	72	58	61	54
GALFP	100	123	161	212	286	127	137	162	172
GAME	27	30	32	36	41	23	28	34	42
PSO+	1.1	3.1	3.4	7.3	19.4	4.9	10.6	2.3	3.8

As shown in Table 1 PSO+ is significantly more efficient than the other algorithms. Our previous PSO algorithm was based on a very different problem formulation and is considerably slower than PSO+. It is worth noting that the running time of the evolutionary algorithms, GAME and GALFP, are much slower than PSO+ in general, but their running time only increases slightly with sequence lengths. This might be because these two algorithms spend a significant amount of time in initialization, independent of sequence lengths.

Next, we compared these algorithms on their performance on challenge problems with varying motif lengths and number of error, while sequence lengths were fixed at 600 bp. The current algorithm outperforms the existing algorithms again on these test sequences (Table 1 right half). Similar to our previous PSO-based algorithm, the running time of PSO+ is relatively independent of motif lengths.

**Figure 2:**
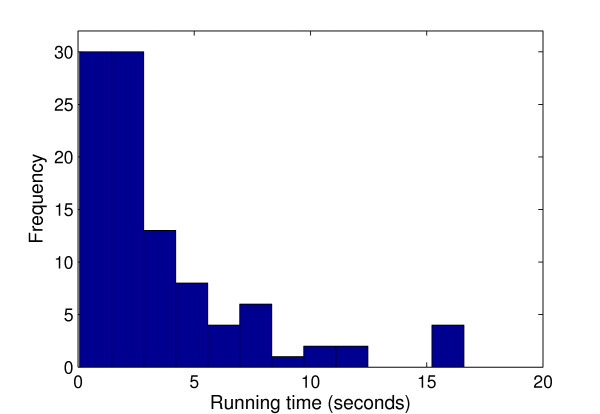
**Running time distribution**. Running time distribution of PSO+ on (15,4)-motif challenge problem (sequence length = 600). Results are based on 100 runs on independent sequences.

Third, we ran PSO+ 100 times on independent sequences, and plotted the running time in Figure 2. As shown, the running time has a long-tail distribution, meaning in some rare cases the algorithm may need an extremely long time to converge. Because of this, the algorithm runs faster than the average running time in most cases. A better strategy for detecting local optima may eliminate some of these rare cases and improve the overall efficiency. The running time results for GALFP/GAME are shown in Additional file 1 Figure S2 and Figure S3.

Finally, we tested our algorithm using synthetic sequences containing gapped motifs, and compared with the existing algorithms. We first generated sequences with embedded gapped motifs, much like the synthetic challenge problems, except that each embedded motif contains some gaps in the middle positions. Only two algorithms, namely, PSO+ and GALFP, can correctly identify these synthesized gapped motifs. It is not surprising to see that the other algorithms, which are not designed to find gapped motifs, failed at these synthetic test cases. Remarkably, GALFP had basically the same accuracy as PSO+. With further investigation, we found that using synthetic data sets with purely random background noise is insufficient to demonstrate the limitation of existing algorithms on finding gapped motifs. This is because gapped motifs can be approximately represented by a PWM with uniformly distributed base frequencies in the gapped positions, and that a motif generated by the synthetic model with a purely random background is either strong enough so that it can be found by both PSO+ and GALFP, or it is so weak that neither algorithm can find it. Therefore, we further generate two additional types of test cases with gapped motifs. Each test case of the first type consists of 20 sequences of length 600, and each sequence contains a gapped motif of length 15 and gap length 5 and a non-gapped motif of length 15. For this type of test cases, GALFP (as well as the other algorithms listed in Table 1) always report the non-gapped motif; while the PSO+ algorithm report the gapped motif as first motif, and the non-gapped motif as the second motif. For the second type of test cases, we generate 30 sequences of length 600. We then generate a gapped motif of length 15 and gap length 5, and embed it on 10 of the sequences, while leave the remaining 20 sequences without any embedded motifs. For this type of test cases, only GALFP and PSO+ can report the correct motif. GALFP only reports a single motif and it has a 91 percent accuracy. PSO+ has an accuracy of 96 percent when only reporting a single motif for each test case, while its accuracy improved to 98 percent with two motifs each test case and 100 percent with eight motifs each test case.

### Experiments on real biological sequences

To test the performance of our algorithm on real biological sequences, we used the same eight representative test cases used in [11], which covered different lengths of motifs and both gapped and non-gapped motifs. In these data sets, some sequences may contain zero or more binding sites. Details of the data sets are available in [11]. Based on these data sets, it has been reported that two genetic algorithms, GALFP [11] and GAME [10], are significantly more accurate than five popular algorithms: MEME [2], Bioprospector (BP) [5], BioOptimizers based on MEME (BOM) and BioOptimizers based on Bioprospector (BOB) [18]. Therefore, we only compared PSO+ with GALFP and GAME directly.

As in [11], we measure accuracy by *precision*, *recall*, and *F-score*. Precision is defined as *c/p *and recall is defined as *c*/*t*, where *c*, *p *and *t *are the number of correctly predicted binding sites, the number of predicted binding sites and the number of true binding sites in the sequences, respectively. The *F-score *combining both *precision *and *recall *is defined as *F *= 2 * Precision * Recall = (Precision + Recall). Table 2 shows the average results of PSO+, GALFP [11] and GAME [10] on 20 runs (the bold entries are the winners). As shown, PSO+ and GALFP have about the same accuracy. PSO+ has the best *F-scores *on 4 of 8 test cases while GALFP has the best *F-scores *on 3 test cases. More in-depth investigation reveals that PSO+ generally has the highest precision (5 out of 8), while GALFP has the highest recall (3 out of 8). This indicates that PSO+ reported less but more accurate binding sites than GALFP.

**Table 2:** Comparisons of average performance on the 8 real datasets, the bold numbers are the best results among three algorithms

	GAME			GALFP			PSO+		
	***Precision***	***Recall***	***F-score***	***Precision***	***Recall***	***F-score***	***Precision***	***Recall***	***F-score***

CREB	0.43	0.42	0.42	0.70	**0.84**	**0.76**	**0.76**	0.68	0.72
CRP	0.79	**0.78**	0.78	0.99	0.73	0.84	**1**	**0.78**	**0.88**
ERE	0.52	0.78	0.62	0.82	0.76	0.79	**0.92**	**0.92**	**0.92**
E2F	**0.79**	**0.87**	**0.83**	0.77	0.85	0.81	0.68	0.7	0.69
MEF2	0.52	0.55	0.53	0.91	0.98	0.95	**1**	**1**	**1**
MYOD	0.14	0.14	0.14	**0.57**	**1**	**0.72**	0.20	0.43	0.27
SRF	0.71	0.86	0.78	0.75	**0.89**	**0.82**	**0.80**	0.56	0.66
TBP	0.81	0.74	0.77	**0.87**	0.87	0.87	0.86	**0.91**	**0.88**

**Figure 3:**
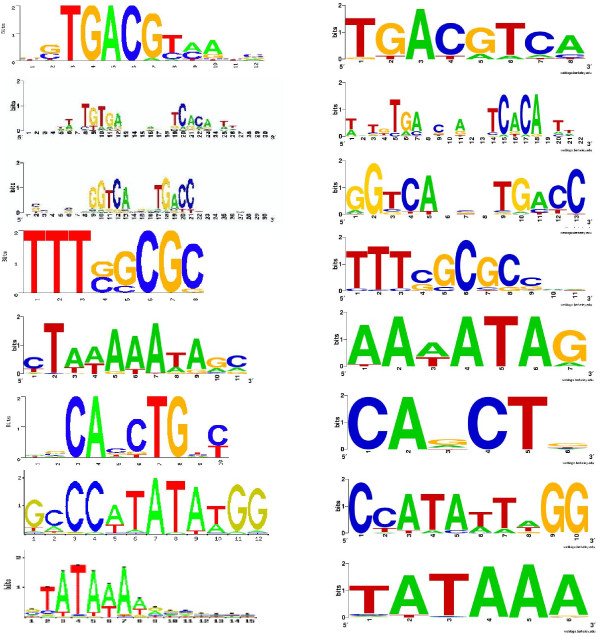
**Experimental results on real motifs**. Left: real motifs; right: motifs found by our algorithm. The motifs are listed in the same order as in Table 2 (CREB, CRP, ERE, E2F, MEF2, MYOD, SRF, TBP).

**Figure 4:**
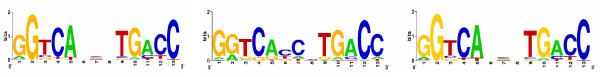
**Logos for the ERE motif**. Left: real motif; middle: motif found by PSO+ with the gap option turned off; right: motif found by PSO+ with the gap option turned on.

Interestingly, two of the eight cases (CRP, ERE) are clearly gapped motifs, as shown in Figure 3. PSO+ won in both cases, especially in ERE (average F-score: 0.92, 0.79, and 0.62, for PSO+, GALFP and GAME, respectively), indicating that the gap model in our algorithm is effective. On the other hand, even though our algorithm has a much larger search space by allowing gaps, its performance on the other six motifs that do not have gaps is still among the best. Figure 4 shows the results of PSO+ on the ERE motif, with or without the gap option. Without the gap option, our algorithm attempts to maximize the total information content of all positions, which results in a motif with relatively uniform information content across all positions. In contrast, with the gap option, our algorithm automatically determines the low-information positions and treats them as gaps, and therefore improves the accuracy of the final result. With the gap option turned on, the Pearson correlation coefficient between the predicted and true motif PWMs is improved from 0.92 (*p *= 10^-22^) to 0.97 (*p *= 10^-33^).

Finally, it takes our algorithm 20 to 60 seconds for each of the 8 test cases. In comparison, the average running time is about 62 seconds for GALFP, and 291 seconds for GAME.

## Conclusions

In this work, we have proposed a novel algorithm for finding DNA motifs based on Particle Swarm Optimization (PSO). Our contributions include a novel modification of the PSO update rule to allow discrete variables, a model to allow gapped motifs, and a simple method to ne-tune the motif when some sequences contain zero or multiple binding sites. Experimental results on synthetic challenge problems as well as real biological sequences show that our method is both more efficient and more accurate than several existing algorithms, especially when gaps are present in the motifs. We are working to finalize our program, which will be freely available to the research community soon.

## Competing interests

The authors declare that they have no competing interests.

## Authors' contributions

CL and JR conceived of the research and designed the study. CL implemented the algorithm and conducted the experiment. CL wrote the paper and JR helped with the manuscript preparation. Both authors read and approved the final manuscript.

## Supplementary Material

Additional file 1 **Supplemental Materials**. Additional figures, tables and discussion. Click here for file

Additional file 2 **Experimental Dataset**. The synthetic data sets and real biological sequences used in the paper. Click here for file
