# Supplementary material for: A particle swarm optimization-based algorithm for finding gapped motifs
Source: BioData Min. 2010 Dec 13;3:9. doi: 10.1186/1756-0381-3-9 (PMC3022572; doi:10.1186/1756-0381-3-9)
Supplement: Additional file 1 — Supplemental Materials. Additional figures, tables and discussion. [file 1756-0381-3-9-S1.DOCX]

Figure S1. Flow Chart of the PSO+ algorithm

Start

restart

Initialize the solution *(current_k_)* for each agent ,*k*

Scan each sequence to find a best match to *current_k_*

Check next agent *current_k+1_*

Calculate the fitness value of *current_k_*

If fitness(current_k_) larger than *Pbest_k_*

N

Y N

No improvement of
*Gbest* in N iterations

Update *Pbest_K_*

If fitness(current_k_) larger than *Gbest*

N Y

Reach the Max number of restarts

N

Y

Update *Gbest*

Y

All agents are finished

Post processing

N

Update all agents by update rule

Check shift

Y

Report the result

Figure S2. Running time distribution of GALFP

Figure S3. Running time distribution of GAME

Table S1. The running time for PSO+ algorithm on separate gaps test cases, with and without turning on the "continuous gap" option.

|  | With continuous gap option | Without continuous gap option |
| --- | --- | --- |
| Mean | 17.50 | 17.14 |
| Median | 17.16 | 16.83 |
| Std | 1.09 | 0.92 |

Table S2. The running time for PSO+ algorithm on continuous gaps test cases, with and without turning on the "continuous gap" option.

|  | With continuous gap option | Without continuous gap option |
| --- | --- | --- |
| mean | 17.33 | 16.78 |
| median | 16.67 | 16.64 |
| std | 1.21 | 0.95 |

Synthetic datasets of gapped motifs

We first tried to generate test cases with gapped motifs in a purely random background. Only two algorithms, namely, PSO+ and GALFP, can correctly identify these synthesized gapped motifs.

We did more experiments, and found that using synthetic data sets with purely random background is not a good way to demonstrate the limitation of existing algorithms on finding gapped motifs. Basically, the synthetic motifs are generated using three parameters: length of motif, length of gap, and number of mismatches from consensus. We found that in a purely random background, a motif is either strong enough so that it can be found by both GALFP and PSO+, or it is so weak that neither algorithm can find it, even with the gap option on. The reason is because the motif signal changes discretely. When the motif generator changes the parameters a little, the signal will change a lot, which causes the problem that motif signal is either too strong or too weak for both algorithms. However, the gap option is effective in the real experiment. Since in the real data, the background information is not purely random and there are many background noises.

To show this, we specially designed some test cases, which contain non-random background noise (non-gapped motifs). We generate a gapped motif and a non-gapped motif, embed them into each sequence, and use the sequences as the input file. In these test cases, if our algorithm does not use the gap option, it will report the noisy motif (non-gapped motif), the same as the GALF-P; but if we turn on the gap option, we can find the real gapped motif.

Additionally, we also generated some test cases where some sequences do not contain motifs. We generate 10 sequences with a gapped motif (which length is 15 and the gap length is 5), add 20 purely random sequences without any embedded motifs, and use these 30 sequences as the input. We run 100 different test cases. GALFP found 91 correct answers. PSO+ reported 96 correct answers as the 1^st^ motif, 2 correct answers as the 2^nd^ motif, 1 correct answer as the 4^th^, and 1 correct answer as the 8^th^.
